# The Role of Smoking Status in Making Risk-Informed Diagnostic Decisions in the Lung Cancer Pathway: A Qualitative Study of Health Care Professionals and Patients

**DOI:** 10.1177/0272989X231220954

**Published:** 2024-01-19

**Authors:** Georgia B. Black, Sam M. Janes, Matthew E. J. Callister, Sandra van Os, Katriina L. Whitaker, Samantha L. Quaife

**Affiliations:** Wolfson Institute of Population Health, Queen Mary University of London, London, UK; Department of Applied Health Research, University College London, London, UK; Lungs for Living Research Centre, UCL Respiratory, University College London, London, UK; Department of Respiratory Medicine, Leeds Teaching Hospitals NHS Trust, St James’s University Hospital, Leeds, UK; Department of Applied Health Research, University College London, London, UK; School of Health Sciences, University of Surrey, Guildford, UK; Wolfson Institute of Population Health, Queen Mary University of London, London, UK

**Keywords:** lung cancer, risk, clinical decision-making, qualitative research

## Abstract

**Background:**

Lung cancer clinical guidelines and risk tools often rely on smoking history as a significant risk factor. However, never-smokers make up 14% of the lung cancer population, and this proportion is rising. Consequently, they are often perceived as low-risk and may experience diagnostic delays. This study aimed to explore how clinicians make risk-informed diagnostic decisions for never-smokers.

**Methods:**

Qualitative interviews were conducted with 10 lung cancer diagnosticians, supported by data from interviews with 20 never-smoker lung cancer patients. The data were analyzed using a framework analysis based on the Model of Pathways to Treatment framework and data-driven interpretations.

**Results:**

Participants described 3 main strategies for making risk-informed decisions incorporating smoking status: guidelines, heuristics, and potential harms. Clinicians supplemented guidelines with their own heuristics for never-smokers, such as using higher thresholds for chest X-ray. Decisions were easier for patients with high-risk symptoms such as hemoptysis. Clinicians worried about overinvestigating never-smoker patients, particularly in terms of physical and psychological harms from invasive procedures or radiation. To minimize unnecessary anxiety about lung cancer risk, clinicians made efforts to downplay this. Conversely, some patients found that this caused process harms such as delays and miscommunications.

**Conclusion:**

Improved guidance and methods of risk differentiation for never-smokers are needed to avoid diagnostic delays, overreassurance, and clinical pessimism. This requires an improved evidence base and initiatives to increase awareness among clinicians of the incidence of lung cancer in never-smokers. As the proportion of never-smoker patients increases, this issue will become more urgent.

**Highlights:**

- Smoking status is the most common risk factor used by clinicians to guide decision making, and guidelines often focus on this factor.
- Some clinicians also use their own heuristics for never-smokers, and this becomes particularly relevant for patients with lower risk symptoms.
- Clinicians are also concerned about the potential harms and risks associated with deploying resources on diagnostics for never-smokers.
- Some patients find it difficult to decide whether or not to go ahead with certain procedures due to efforts made by clinicians to downplay the risk of lung cancer.
- Overall, the study highlights the complex interplay between smoking history, clinical decision making, and patient anxiety in the context of lung cancer diagnosis and treatment.

Lung cancer is the third most common cancer in the United Kingdom, accounting for approximately 13% of all new cancer cases each year.^1,2^ UK survival rates have not shown much improvement over the past 40 y; the 5-y relative net survival for lung cancer is below the European average. Its high incidence combined with poor survival rates make it the leading cause of cancer death in the United Kingdom.^1^ Never-smokers (people who have smoked less than the equivalent of 100 cigarettes in their lifetime)^3^ make up 14% of the lung cancer population. To put this into perspective, when measured as a separate cancer, lung cancer in never-smokers is the eighth most prevalent cause of cancer-related death.^2^

Diagnosing lung cancer in the United Kingdom often begins in primary care with a chest X-ray when patients present with respiratory symptoms of concern.^4^ An abnormal chest X-ray will provoke referral to secondary care, and further imaging investigations may include a computed tomography (CT) scan or a positron emission tomography–CT, followed by a diagnostic biopsy of the primary mass (often with a CT-guided biopsy) or secondary deposits using endobronchial ultrasound-guided transbronchial needle aspiration, endoscopic ultrasound fine-needle aspiration, or ultrasound or CT-guided biopsy. These tests are associated with potential harms from radiation or clinical complications.^5–7^ However, there are also harms associated with diagnostic delays such as poorer survival rates, treatment delays, and cancer stage at diagnosis.^8–11^ National Institute for Health and Care Excellence (NICE) guidance for lung cancer states that health care professionals should “choose investigations that give the most information about diagnosis and staging with the least risk to the person.”^12^

This is an example of risk-informed decision making: a deliberative process that uses a set of known parameters together with other information to guide a decision. The process incorporates human judgment, rather than merely relying on technical information. This is particularly relevant to scenarios in which the decision maker lacks information and also in which the decision is intrinsically subjective with competing priorities.^13^ Risk is a combination of both the severity and likelihood of an unwanted outcome.^14^

Smoking puts individuals at greatly increased likelihood of lung cancer as well as cancer recurrence and mortality.^15,16^ Therefore, smoking status is a key piece of information for clinicians weighing the potential harms and benefits to patients of further invasive tests.^7,17^ These tests may result in overdiagnosis or cause psychological harms for the patient.^18,19^ The likelihood of lung cancer is combinatorial, based on causative factors such as age, family history, smoking history, and presenting symptoms.^14,20^ Prognostic information also depends partly on smoking history in terms of the predisease health of the patient and the mechanism of oncogenic activation.^20^ Our rapid review of evidence about lung cancer diagnosis for patients who never smoked and patients with a smoking history concluded that evidence about diagnostic harms and benefits for never-smoker patients specifically is lacking,^21^ making it difficult for clinicians to make risk-informed decisions.

This article reports data from the PEARL study (Patient Experience of symptoms, help-seeking And Risk factors in Lung cancer in never, current and former smokers). The overall objective of the study was to explore health care professionals’ and lung cancer patients’ perspectives of lung cancer diagnosis (investigations, diagnosis, and patient support needs), identifying differences by smoking status (never, former, or current smoker). The aim of this article is to understand how smoking history affects risk-informed decision making in the lung cancer diagnostic pathway.

## Methods

Our study methods relate to the decisions made by practitioners in both primary and secondary care, which we refer to as the “lung cancer pathway.” The UK health care system operates through a taxation and national insurance model that is free for patients at the point of contact, which creates a resource-gatekeeping role for the general practitioner. All patients must seek care initially through primary care, where a general practitioner can either refer a patient directly for a chest X-ray or make an urgent suspected cancer referral to a respiratory multidisciplinary team in secondary care.

These methods are reported in accordance with the COnsolidated criteria for REporting Qualitative research (COREQ) checklist.^22^ This was a qualitative interview study primarily using semistructured telephone interviews with clinicians from across the lung cancer diagnostic pathway including radiology, nursing, surgery, respiratory medicine, and general practice. Data from patient interviews were also included, which covered their experiences and perspectives of their diagnostic journey. Clinicians were asked about their experiences of working with never-smokers and how they make diagnostic and management decisions. The study was considered and approved by the UCL Research Ethics Committee (project ID 17701/001).

### Sampling and Recruitment

#### Health care professionals

Ten clinicians involved in the diagnosis/care of lung cancer patients were recruited using snowballing through our clinical collaborators, including general practice and respiratory medicine settings in England (Table 1).

**Table 1:** Health Care Professional Characteristics

Characteristics	N
Sex	
Male	3
Female	7
Profession	
Radiographer	1
Specialist nurse	3
Thoracic surgeon	1
General practitioner	3
Respiratory consultant	2

#### Patients

Individuals from across the United Kingdom who received a primary lung cancer diagnosis in the previous 12 mo were recruited by a specialist recruitment company (Taylor McKenzie Ltd; TM). Individuals were excluded if their recent diagnosis was a recurrence of a previously treated lung cancer disease (>12 mo ago) but could be included if they had had other cancers in the past. TM invited individuals who previously consented to be contacted about research as part of their commercial database and reached out to support groups, charities, and patient organizations through social media. TM explained the rationale for the study and answered any preliminary questions about the research and then introduced them to the researcher via e-mail. SvO contacted potential participants to establish a relationship and complete the interview. We interviewed patients with different smoking histories, but this study reports the data from participants who had never smoked (*N* = 20; Table 2).

**Table 2:** Patient Characteristics

Characteristics	N
Sex	
Male	5
Female	15
Ethnicity	
White British	18
Asian British	2
Age	
Average	51.55
Range	35–68
Significant physical comorbidity	7
Average No. of symptoms at first presentation to primary care	1.2
Time since diagnosis	
Up to 3 mo	4
3–6 mo	10
6–9 mo	2
9–12 months	3
Preferred not to say	1

#### Data collection

Participants were interviewed by SvO, an experienced female qualitative researcher with a background in psychology who has no specialist clinical knowledge of lung cancer. SvO contacted participants via telephone after providing verbal audio-recorded consent. Semistructured interview discussion guides (see Appendix 1) were developed specifically to address the aims of the study, drafted by the study qualitative researchers (GB and SvO), and revised following feedback from patient representatives, academics, and clinicians. Patient interviews explored patients’ perceptions in relation to lung cancer risk and symptoms, their decision to visit primary care about their symptoms, and their experiences of the diagnostic pathway.

All interviews were conducted by SvO, a qualitative researcher experienced in health research who has no specialist clinical knowledge of lung cancer. Interviews were audio-recorded and transcribed verbatim. Participants were not sent their transcripts for comment.

#### Data analysis

Framework analysis was used to process the interview data using Microsoft Excel software.^23^ Initially, 2 researchers read the transcripts and discussed emergent ideas. Two separate coding frameworks were developed, 1 for patients and 1 for health care professionals focusing on issues at different points in the lung cancer diagnostic pathway (symptom appraisal, help-seeking, diagnosis) following the Model of Pathways to Treatment by considering patient factors, health care system and provider factors, and disease factors.^24^ All interviews were systematically coded into these frameworks while taking note of unusual or prominent quotations. These coding frameworks were then used by all the authors to consider the issue of risk-informed decision making. The current analysis is mainly derived from the health care professional interviews. We have published our findings in relation to the patient data elsewhere.^25^ As part of our original analysis, extracts from patient interviews with people who have never smoked were identified that related to harms associated with decision making around diagnostic testing. These are also reported in this article.

## Findings

Our results suggest that making a risk-informed decision about tests and investigations for never-smokers in the lung cancer diagnostic pathway is challenging for clinicians. A variety of different strategies are used to make risk-informed decisions in conjunction with information about the patient. Table 3 outlines the types of information used in each strategy. Guidelines and risk tools rely on smoking history, symptom information, and imaging results to inform diagnostic decisions. However, in the absence of smoking history, clinicians reported more reliance on heuristics or “rules of thumb” to weigh up potential harms and benefits, which relied on gut feeling, patient health state, and patient self-advocacy. The themes below explain how clinicians make risk-informed diagnostic decisions in the lung cancer pathway. Patient data are presented to consider how these decisions were received.

**Table 3:** Health Care Provider–Reported Use of Patient Information and Risk Tools to Make Risk-Informed Decisions in the Lung Cancer Diagnostic Pathway

		Patient Information Used					
		Smoking History	Symptoms	Imaging	Gut Feeling	Patient Health State	Patient Self-Advocacy
Strategies for making risk-informed decisions	Guidelines^a^	✓	✓	✓			
	Risk scores	✓	✓	✓			
	Heuristics	✓	✓	✓	✓	✓	✓

a National Institute for Health and Care Excellence lung cancer guidelines suggest that all patients receive a chest X-ray for initial evaluation, unless they are aged >40 y with unexplained haemoptysis.^26^ Since 2015, the guideline requires patients to have 2 indicative symptoms if they have never smoked but only 1 if they are a current or former smoker.

### Clinicians Use Guidelines and Tools to Make Risk-Informed Decisions

Our clinician participants reported that few of their decisions about lung cancer investigation were not risk informed by smoking status. They drew on guidelines and scoring systems to justify this practice:

And even the NICE guidance says you only have to have half the symptoms if you’re a smoker . . . you have more symptoms if you’re a nonsmoker. By definition it’s not quite as high up our list of concerns. (HCP5, general practitioner)

Similarly, in secondary care, clinician participants reported using risk-scoring systems such as the Herder score that calculate the likelihood of malignancy and include smoking status as part of the calculation. This score was a key part of their decision making about further invasive tests and exposure to radiation-emitting imaging:

If they’re nonsmokers then that would automatically change some of their risk of malignancy calculation. So whenever we come across a tumor, you probably have heard of this before, but when we come across the tumor, if we don’t have a tissue diagnosis we have to calculate the probability that there is a lung cancer, and smoking is one of the prime markers for that. . . . The Herder model that [calculates the] chance of malignancy, and that’s assessed, should we go ahead with more investigations? Should we just go ahead and treat, or is it not a lung cancer at all? And so, if you don’t smoke that automatically reduces your risk of malignancy. (HCP7, consultant thoracic surgeon)

Supporting this, several patient participants were aware that they did not meet the criteria for referral due to their smoking status, which presented a barrier to further testing. One participant mentioned that they were repeatedly asked about smoking status when their symptoms did not resolve but that no further action was taken:

All I kept getting asked was, “Do you smoke, have you been abroad recently?” I went, “No I don’t smoke, I’ve never been a smoker and I haven’t been abroad for a while, the last place I went was Tenerife the year before.” So I kept getting asked the same question, I kept getting different doctors at this point and then they prescribed me another antibiotic . . . I kept ringing up and saying, “Nothing’s happening.” So they gave me more antibiotics at which point they were really unhelpful and said to me, “Well there’s not really much else we can do about a cough, it can just take time, we can give you more antibiotics if you want.” (Participant 30)

Despite repeated questioning about smoking status, this participant was asked very few questions about other risk factors including her worsening symptoms. There was an admission by a minority of clinician participants that they did not always ask patients about risk factors included in the NICE guideline for lung cancer/mesothelioma other than smoking history, such as passive smoking history or asbestos exposure^27^:

I think we’re not so good at asking all of that, about passive smoking in terms of where they worked or in the home . . . I very rarely go on to say, “Do you live with a smoker?” which actually would be pretty significant . . . in terms of diagnosis and this cross stratification, that’s probably something we don’t do very well. (HCP9, general practitioner)

### Clinicians Supplement Guidelines with Additional Heuristics to Make Risk-Informed Decisions

Some clinicians reported using their own thresholds or heuristics to make risk-informed radiologic decisions for patients without a smoking history (see Table 4).

**Table 4:** Information Used to Make Radiologic Decisions for Patients without a Smoking History as Reported by Clinicians

Radiologic Decision	Information Used for Risk-Informed Decision	How Does Smoking History Affect the Threshold for Decision?
Order chest X-ray	Severity of symptoms Duration of symptoms	Threshold set at more severe symptoms or longer duration for patients without smoking history
Make abnormal chest X-ray determination	Size of identified abnormality	Threshold set higher if abnormality is small for patients without smoking history
Request computed tomography scan	General practitioner/radiologist gut feeling	Threshold the same regardless of smoking history

Sometimes this resulted in new thresholds for radiologic decisions, such as requesting a chest X-ray after 6 wk for a never-smoker rather than 3 wk:

What I would expect is that you might have a higher threshold for doing the chest x-ray after a cough for the 3 weeks. You would still do it because you would be interested in other diagnoses as well but you might have a lower threshold . . . you might have a higher threshold for doing a chest X-ray slightly . . . you might do the chest X-ray at 6 weeks, not at 3 weeks. (HCP6, general practitioner)

Similarly, a radiographer admitted that they might be more cautious about patients with a smoking history, particularly when it came to considering a potential false-negative X-ray and suggesting a follow up CT scan:

If you get a smoking history, it does probably tip your balance of the diagnosis you’re making towards being more cautious, . . . because as someone who reports the X-rays, you’re aware of the limitations of the X-ray and now with low dose, low dose is a relative thing, but with low-dose CT scans, and you don’t always have to use intravenous contrast mediums as part of the scan, the threshold to referring for CT is relatively low. (HCP1, radiographer)

Several clinicians indicated that decisions were easiest with never-smokers when the symptoms were obvious or severe:

And if someone comes in, let’s say, with hemoptysis, you’re gonna do an X-ray regardless, but you don’t even have to ask them if they smoke or not, you’re gonna do that investigation. So some of them could have come through and I’m not realizing they didn’t smoke, if that makes sense, ‘cause you just didn’t have to ask the question, ‘cause you’re already on the kind of pathway. (HCP5, general practitioner)

This was also the case for decision making in secondary care, where the size of any abnormalities was an important factor, whereas vague symptoms were assigned less weight:

And so my threshold for an abnormal on a never-smoker is probably high for the subtle things, if it’s a big blob, round thing, then it’s the same threshold, if it’s I’m not sure whether it’s a bit of rib with a bit of blood vessel, a bit of shoulder blade in a never-smoker, your threshold for abnormality is probably higher than if they have established COPD . . . your threshold for calling something equivocal is lower than a never-smoker with the same history of weight loss. (HCP1, radiographer)

Subjective decision making was justified by several clinician participants under the terminology of “gut feeling,” which they related to having a reason to be concerned (or not) depending on criteria beyond guidelines or risk calculation tools:

So definitely with gut feeling is not something we ignore, especially when it comes from the radiologist. If the radiologist’s gut feeling, on the appearance of the tumor from the scan, is considered almost as highly as any score calculation. So if they are genuinely concerned then we do further investigations regardless of the Herder score. If the scoring system was coming out saying the chance of this being malignancy is 1% or 2% and the radiologists were still worried we would, at least, organize another CT. We don’t just let it go. (HCP4, consultant thoracic surgeon)

Two clinician participants noted professional differences in judgment about the likelihood of lung cancer for never-smoker patients. It was suggested that diagnosticians and respiratory clinicians could have different heuristics for judging risks for never-smokers than other professionals would, based on their greater experience of lung cancer in never-smokers helping to challenge this tobacco-centric heuristic in decision making:

I think the people who work in lung cancer they have awareness. I think it’s probably more the problem if people present in nonrespiratory, I think all respiratory doctors probably have that in mind as a differential diagnosis. It’s probably more the problem of nonrespiratory because if they see a lesion on the lung in a 30-year-old they probably would think of lots of other things. But not so much that that could be lung cancer as well. (HCP8, consultant)

Guidelines that were highly risk-informed by smoking status were supplemented or circumvented by subjective risk judgments that were also dependent on smoking status and symptom severity. In other words, never-smoker patients with low-risk symptoms could be less likely to be referred or investigated even if they met the guidelines.

### Clinicians Consider Potential Harms to the Patient to Make Risk-Informed Decisions

A key factor in risk-informed decision making was the likelihood of harms to the patient. Three types of harm were identified by clinicians: opportunity costs in a climate of scarce resources, physical harms, and psychological harms. Table 5 reports quotations about how these potential harms affected decision making and other behaviors. In contrast, patients reported primarily process harms as a consequence of HCP risk-informed decisions such as delays and miscommunications.

**Table 5:** Clinician- and Patient-Reported Harms in Diagnostic Decision Making for Potential Lung Cancer

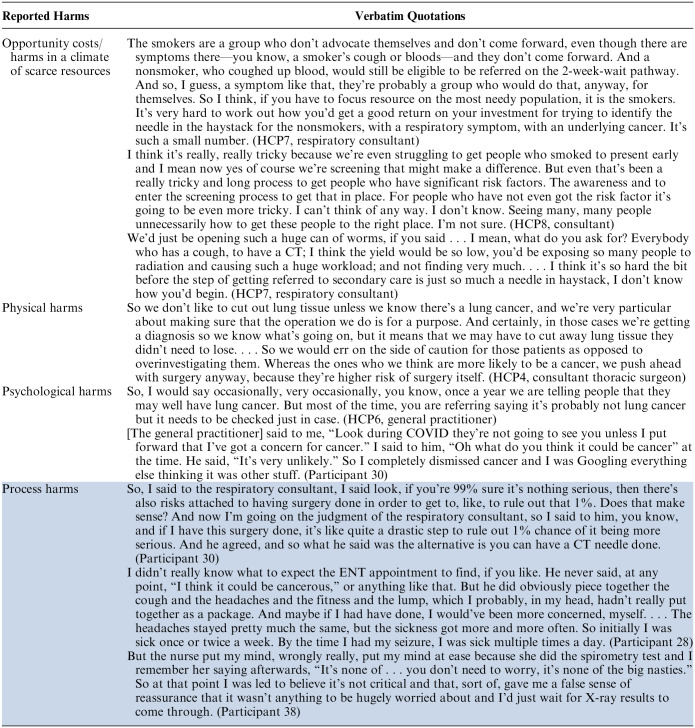

#### Opportunity costs/harms in a climate of scarce resources

Some clinicians reflected that never-smokers were both a more health literate and socioeconomically advantaged population and at much lower risk of cancer. Therefore, any potential harms or risks associated with deploying scarce resources on diagnostics for this group were justified, particularly as inclusion in pathways for red flag symptoms was viewed to be equivalent regardless of smoking status. Notably, in our interviews, clinicians did not reflect on the impact of other risk factors such as passive smoking or occupational exposures.

Underlying these views was the idea that never-smokers with lung cancer were a very small minority of the patient population that deprioritized their needs and right to resources in comparison with smokers. Some clinicians were pessimistic about improving early diagnosis for never-smokers, particularly given the challenges in identifying which of those presenting were at risk of lung cancer and because getting the pathway right for smokers was already very difficult. This was sometimes characterized by concerns about increasing existing staff workloads.

#### Physical harms

Clinicians reported that they made decisions about diagnostic tests and procedures based on their judgment of likely physical harm to the patient. Harms were characterized as “overinvestigating” and could include complications caused by a biopsy needle, unnecessary surgical excision of healthy tissue, or radiation to the patient (see Table 5). However, the decision to proceed with investigations was made more easily for patients with a smoking history. Another potential harm of overinvestigating was the associated increased workload and would not yield gains in terms of cancers diagnosed early.

#### Psychological harms

Clinicians reported that they preferred not to cause unnecessary worry for never-smoker patients, in line with their own lower expectations of these patients actually having lung cancer, and so would emphasize their lower likelihood of cancer, presenting further tests as “rule-out” rather than “rule-in.” The 3 general practitioners we interviewed agreed that increasing awareness about lung cancer for never-smokers could increase anxiety. One general practitioner reported telling patients that they “probably” did not have lung cancer at the point of referral in order to reduce the potential psychological harm.

Even after their symptoms had persisted for some time, patient participants reported being told that they were still unlikely to have lung cancer. Several patients experienced reassurance by multiple clinicians that their symptoms were unlikely to be a result of lung cancer.

#### Process harms

Patient-reported harms were related to process issues such as delays and difficulty making decisions (see Table 5), rather than experiencing anxiety about cancer. Patients had low expectations about the likelihood of cancer, caused both by preexisting beliefs^25^ and HCP strategies to reduce psychological distress, as described in the previous theme. Patients were falsely reassured that they were unlikely to have cancer. In some cases, this led to delays in chasing up tests or reconsulting with a clinician. A minority of patients found it harder to make informed decisions about investigations, for example, whether or not to have exploratory surgery if their likelihood of cancer was very low.

## Discussion

This is, to our knowledge, the first qualitative study to explore how diagnostic decisions for potential lung cancer are made by health care professionals and how these are experienced by patients, based on the patient’s smoking history. Health care professionals rely on formal guidelines and risk-calculation tools, which place more weight on smoking status than other potential risk factors. Therefore, in the absence of a smoking history, these tools may be less valuable for differentiating high- from low-risk patients, leaving clinicians to incorporate their own heuristics to make judgments about never-smokers, for example, the severity of their symptoms and “gut feelings.” Potential harms were considered in making diagnostic decisions, such as the risk of causing physical harm, squandering scarce resources at an opportunity cost to patients with a smoking history, and causing unnecessary anxiety to patients. However, efforts to reassure never-smokers of their lower risk of cancer exacerbated process harms such as delay and difficulty navigating care.

### Relevance to Other Published Work

#### Risk-informed decision making in lung cancer diagnostic pathways

Other studies have recognized that decision making about potentially harmful lung cancer investigations for patients at low risk is particularly challenging for clinicians, regardless of smoking history.^28^ Supplanting guidelines and tools with clinical judgment is inherent in risk-informed decision making, and prior research suggests that this is common. A survey study revealed that general practitioner adherence to guidelines is relatively low in the United Kingdom compared with other comparable nations.^29^ Similarly, an audit of primary care in Scotland found that about 10% of patients referred for suspected cancer investigations were not guideline compliant; however, a large proportion of these patients were diagnosed with cancer (8.8% for lung).^30^ This justifies the use of additional heuristics and “gut feeling” while making diagnostic decisions (particularly in primary care) but raises concern about clinical situations in which the guideline does not have enough detail or definitive suggested actions. This may be particularly relevant to never-smokers, for whom there is no additional advice in the NICE guidance nor criteria effective in differentiating those with increased risk.^27^

One potential limitation to the current guidelines and risk tools in practice are that they do not contain much detail nor risk criteria that relate specifically to the etiology or signs of disease in never-smokers. Several risk models for lung cancer in never-smokers have been developed but mainly focus on static features such as gender and age.^31^ None of these models incorporate symptom information such as those derived for primary care risk calculation including smokers.^32^ Future tools may incorporate environmental risk factors such as pollution^33,34^; however, our results suggest that clinicians may be less likely to ask patients about environmental factors, limiting the utility of such tools. Even if they did, there is some evidence to suggest that patients may be unaware of their own exposure,^35^ and environmental exposures are difficult to quantify and measure.

We suggest that clinicians would benefit from enhanced guidance and methods for risk differentiation specifically tailored to never-smokers, in order to mitigate diagnostic delays, overreassurance, and clinical pessimism. The current reliance on heuristics, gut feelings, and subjective assessments highlights the limitations of existing referral tools in effectively addressing the needs of this patient population.

#### Avoiding psychological harms to patients by emphasizing never-smokers’ lower risk

It is often advised that clinicians should avoid causing patients unnecessary anxiety around cancer investigations while also avoiding inappropriate reassurance.^36^ Our results suggest that clinicians emphasize the low likelihood of lung cancer and where possible frame diagnostic tests using rule-out language, particularly to never-smoker patients. There is evidence from previous studies that this is a strategy used across all cancer pathways. However, our results also suggest that never-smokers who are diagnosed with lung cancer could find it more difficult to make risk-informed decisions about their care due to clinician communication. For example, one study showed that general practitioners avoid giving information leaflets at the point of referral because they contain information about cancer.^37^ In another qualitative study, general practitioners did not want to name too many cancer signs and symptoms as part of safety netting advice, as this could cause anxiety.^38^ Other studies suggest that patient anxiety in the lung cancer diagnostic pathway is ubiquitous, but clinicians should be reassured that patients also have their own coping strategies (e.g., drawing on social network).^39^

Our study adds particular value in considering how the risk-informed decision-making process may be contributing to patient-clinician communication about risk; without better tools for differentiating risks for never-smokers, clinicians may be falsely reassured and transmit this to their patients. Our results also suggest that efforts to avoid psychological harms for patients may have unintended consequences in terms of delayed diagnosis, which leads to poorer outcomes.^40,41^

### Study Limitations

This study had several limitations. We used a relatively small sample size of health care professionals, which might have affected the generalizability of the findings. In addition, our sample was predominantly professionals from secondary care. This may have emphasized certain viewpoints based on the selection of participants who are particularly interested in never-smoker patients and who were willing to take part. Our study included health care professionals from different localities in England; however, they were mainly concentrated in urban, metropolitan areas, and this might have affected their perspectives. In addition, our patient sample was limited to those diagnosed with lung cancer, which might have led to different perspectives about the harms of overinvestigation compared with patients who underwent diagnostic tests and did not have lung cancer. Finally, the study is limited by relying on interview data and self-reported practice; future studies would benefit from observational components to record actual behaviours. Therefore, our study provides an initial exploration of these issues; however, we believe this to be the first of its kind and thus a useful starting point for exploring future decision-making interventions and strategies for never-smokers and other low-risk patients.

### Implications for Policy and Research

#### Policy

Guidelines for never-smoker patients could explore different thresholds for imaging and referral with clinical stakeholder input. The threshold for chest X-ray in the 2015 NICE guideline for lung cancer could remove all reference to smoking status (as per the 2008 guidelines).^42^ Chest X-ray is a cheap, low-radiation, and widely available test and provides valuable risk information.^43,44^ Risk tools and guidelines could be developed specifically to consider whether to refer symptomatic never-smoker patients with a negative chest X-ray. Policies concerned with patient communication at the point of referral and testing should include warnings not to overly reassure or omit the risk of cancer to patients. This should be supported by reminders to ask patients about nonsmoking risks during consultations, including guidance on assessing these (e.g., occupational exposures). Some clinicians may also require further guidance about nonsmoking risk factors.

#### Research

Studies should develop and co-design evidence-based messaging and communication training that promotes understanding at each stage of the diagnostic pathway while also optimizing psychological well-being. Strategies that promote perceptions of agency and control over the diagnostic pathway and improving lung cancer outcomes are likely to be promising. Research would be valuable that prioritizes shared decision making and optimal communication for patients at lower risk where guidelines are unclear.

Due to the limitations of current referral tools, health care professionals rely on their gut feelings, heuristics, and experience. Further research should aim to increase the precision of risk prediction tools and tests for lung cancer in patients who have never smoked, by improving understanding of the underlying etiology and symptom trajectory as well as valid and reliable ways to measure risk exposures.

## Conclusions

Tobacco smoking history is a dominant factor in objective and subjective risk assessments for diagnostic testing for potential lung cancer, particularly where patients present with ambiguous signs and symptoms. Clinicians would benefit from improved guidance and methods of risk differentiation for never-smokers to avoid diagnostic delays, overreassurance, and clinical pessimism. This ultimately relies on an improved evidence base and initiatives to improve awareness among some clinicians of the incidence of lung cancer in never-smokers. This issue will become more urgent as this patient group increases in proportion to smokers year after year with the success of smoking cessation programs.^45^

## Supplemental Material

sj-docx-1-mdm-10.1177_0272989X231220954 – Supplemental material for The Role of Smoking Status in Making Risk-Informed Diagnostic Decisions in the Lung Cancer Pathway: A Qualitative Study of Health Care Professionals and Patients Supplemental material, sj-docx-1-mdm-10.1177_0272989X231220954 for The Role of Smoking Status in Making Risk-Informed Diagnostic Decisions in the Lung Cancer Pathway: A Qualitative Study of Health Care Professionals and Patients by Georgia B. Black, Sam M. Janes, Matthew E. J. Callister, Sandra van Os, Katriina L. Whitaker and Samantha L. Quaife in Medical Decision Making

sj-docx-2-mdm-10.1177_0272989X231220954 – Supplemental material for The Role of Smoking Status in Making Risk-Informed Diagnostic Decisions in the Lung Cancer Pathway: A Qualitative Study of Health Care Professionals and Patients Supplemental material, sj-docx-2-mdm-10.1177_0272989X231220954 for The Role of Smoking Status in Making Risk-Informed Diagnostic Decisions in the Lung Cancer Pathway: A Qualitative Study of Health Care Professionals and Patients by Georgia B. Black, Sam M. Janes, Matthew E. J. Callister, Sandra van Os, Katriina L. Whitaker and Samantha L. Quaife in Medical Decision Making

sj-pdf-3-mdm-10.1177_0272989X231220954 – Supplemental material for The Role of Smoking Status in Making Risk-Informed Diagnostic Decisions in the Lung Cancer Pathway: A Qualitative Study of Health Care Professionals and Patients Supplemental material, sj-pdf-3-mdm-10.1177_0272989X231220954 for The Role of Smoking Status in Making Risk-Informed Diagnostic Decisions in the Lung Cancer Pathway: A Qualitative Study of Health Care Professionals and Patients by Georgia B. Black, Sam M. Janes, Matthew E. J. Callister, Sandra van Os, Katriina L. Whitaker and Samantha L. Quaife in Medical Decision Making
