# Supplementary material for: The Role of Smoking Status in Making Risk-Informed Diagnostic Decisions in the Lung Cancer Pathway: A Qualitative Study of Health Care Professionals and Patients
Source: Med Decis Making. 2024 Jan 19;44(2):152–62. doi: 10.1177/0272989X231220954 (PMC10865750; doi:10.1177/0272989X231220954)
Supplement: sj-docx-1-mdm-10.1177_0272989X231220954 – Supplemental material for The Role of Smoking Status in Making Risk-Informed Diagnostic Decisions in the Lung Cancer Pathway: A Qualitative Study of Health Care Professionals and Patients [file sj-docx-1-mdm-10.1177_0272989X231220954.docx]

**Interview Topic Guide – HCP interviews**

**Patient Experience of symptoms, help-seeking and risk factors in lung cancer.**

**This document is intended to be a guide.  The following topics/questions/prompts are not exhaustive and the researcher may probe or follow the participants’ line of interest as is appropriate for the purpose of this study.**

**Introduction to study**

- Introduce self and briefly explain project.
- Provide all potential participants with a copy of participant information sheet
- Answer any questions and explain process.
- Remind participants of their rights, including confidentiality, right to withdraw, etc.

**After full explanation of project has been given, if participant says they will take part:**

- Ask participant to read and sign participant consent form.
- Participants in telephone interviews will be asked to verbally confirm they agree to each of the statements on the consent form. Their verbal consent will be audio-recorded.

**Interview**

1. What is a typical day in your work
2. What is your involvement in diagnosis and treatment?
3. How many patients do you typically look after? Diagnose daily/weekly/monthly?
4. In your experience how many never smokers?
5. Is there anything about never smokers that makes them different?
   1. Symptoms
   2. How are they diagnosed
   3. How do they respond
   4. Treatment
   5. Support they require/receive
   6. Specific challenges, how are they different from smokers/ex-smokers?
   7. What is your experience about causes of cancer among never smokers
6. Stigma?
   1. How do you think this affects never smokers?
   2. Anything you (can) do about this?
   3. What do patients say about this?
