# Supplementary material for: The Role of Smoking Status in Making Risk-Informed Diagnostic Decisions in the Lung Cancer Pathway: A Qualitative Study of Health Care Professionals and Patients
Source: Med Decis Making. 2024 Jan 19;44(2):152–62. doi: 10.1177/0272989X231220954 (PMC10865750; doi:10.1177/0272989X231220954)
Supplement: sj-docx-2-mdm-10.1177_0272989X231220954 – Supplemental material for The Role of Smoking Status in Making Risk-Informed Diagnostic Decisions in the Lung Cancer Pathway: A Qualitative Study of Health Care Professionals and Patients [file sj-docx-2-mdm-10.1177_0272989X231220954.docx]

**Interview Topic Guide**

Patient Experience of symptoms, help-seeking and risk factors in lung cancer.

This document is intended to be a guide. The following topics/questions/prompts are not exhaustive and the researcher may probe or follow the participants’ line of interest as is appropriate for the purpose of this study.

**Introduction to study**

- Introduce self and briefly explain project.
- Provide all potential participants with a copy of participant information sheet
- Answer any questions and explain process.
- Remind participants of their rights, including confidentiality, right to withdraw, etc.

**After full explanation of project has been given, if participant says they will take part:**

- Ask participant to read and sign participant consent form.
- Participants in telephone interviews will be asked to verbally confirm they agree to each of the statements on the consent form. Their verbal consent will be audio-recorded.

**Interview**

Structured questions to map the diagnostic pathway.

1. Which signs/symptoms did you experience before you went to the GP?
2. How did the symptoms develop over time?
3. When did you first see the GP about these symptoms?
4. How did your GP visit go?

Prompts

- 1. What happened during the visit?
  2. Did they do any investigations?
  3. Did they arrange any tests?
  4. Did you go back for further visits?
  5. How many visits?
  6. Over how long a period?
  7. Did you see any other healthcare professionals?

1. Who diagnosed you?

Prompts

- 1. When did you receive diagnosis?
  2. Who diagnosed you?
  3. Where were you diagnosed?
  4. What happened after your diagnosis?

Semi-structured interview questions to explore symptom experience and interpretation, help-seeking, illness schemas, experience of primary and secondary diagnostic and care pathways, and social and life history prior to diagnosis. The interview discussion guide will include the below topics, but will not be finalised until after phase 1 (evidence synthesis) has been completed.

Interview topics:

1. Could you tell me a bit more about the signs or symptoms you were experiencing?
2. When did you first notice changes?

Prompts

- - What made you notice?
  - What was happening at the time?
  - Who noticed them?
  - At that time, what did you think was causing it?
  - Have your thoughts about this changed over time?
  - If so, how?
  - Did you look up any information about the symptoms?
    - Internet / family / friends / health care professional
  - Did you talk to anyone else about the symptom?
    - How did you feel about telling others about your symptoms? **Stigma**
    - Do you think it’s a common symptom?
  - What did you family/friends think about your symptoms? **Stigma**
    - Other people?
    - Did they encourage you to seek help?
    - Did you trust their judgements?

1. How did you react the first time you noticed the symptom(s)? Move to question 5 if GP is mentioned.
2. What made you decide to visit the GP?
3. How long was it before you decided to see the GP/make an appointment?

Prompts

- 1. What influenced your decision?
  2. Were there any personal reasons that kept you from going to see the GP? **Stigma**
  3. Would it have affected other people if you went to the GP? Would anyone have said anything to you?
  4. Some people say they feel a bit embarrassed, would you be like that? **Stigma**
  5. Some people worry they are wasting GP time, would you be like that?

1. Was it easy/simple to see the GP?
   1. If not, why?

Prompts

- - 1. Difficult to get to?
    2. Interfere with work/other priorities?
    3. Getting an appointment?

1. How would barriers that were mentioned be overcome?

Prompts

- 1. Is this something that someone else could have helped you with?
  2. Why them? How could they have helped?
  3. Would it be made easier if something were changed in your community?

1. How did that first GP visit go?

Prompts

- 1. What happened?
  2. How did you feel during the visit?
  3. And after?
  4. Were you concerns dealt with/what was the outcome of the visit?
  5. What did you understand about the symptoms after the visit?

1. What happened next?

Prompts

- 1. Probe on experiences in relation to further visits to GP (did they see the same GP, or not? Why/why not?).
  2. Were you referred? (probe who referred them, why, to whom were they referred, experiences, how long did it take, etc.)
  3. Who diagnosed you in the end? (probe experiences about the diagnosis, how long after first visit, what did they say, did you understand what was said, how did you feel, did you talk to family/friends? Did you receive support? Etc).

1. How soon after diagnosis did you start treatment?

Prompts

- 1. Were there delays?
     1. What were they caused by?
     2. Do you think this delay has had a negative impact on you?
     3. What could have prevented the delay?
  2. How is the treatment going?

**Close and debrief**

- Thank you
- Explain what will be done with information
- Remind where contact details can be found
- Check participant is happy and answer any questions.
